# NKp46 recognizes the hyphal form of Candida albicans and mediates protective antifungal immunity

**DOI:** 10.1016/j.isci.2025.113556

**Published:** 2025-09-12

**Authors:** Mingdong Liu, Ahmed Rishiq, Yoav Charpak-Amikam, Fubin Li, Ofer Mandelboim

**Affiliations:** 1 Department of Immunology and Cancer Research, Hebrew University-Hadassah Medical School, Jerusalem, Israel; 2 Shanghai Institute of Immunology, Faculty of Basic Medicine, Shanghai Jiao Tong University School of Medicine, Shanghai 200025, China; 3 The Concern Foundation Laboratories at the Lautenberg Center for Immunology and Cancer Research, Hebrew University Medical School, IMRIC, Jerusalem, Israel

**Keywords:** Immune response, Mycology

## Abstract

*Candida albicans* is an opportunistic fungal pathogen whose ability to switch between yeast and hyphal forms is central to its virulence. Natural killer (NK) cells are critical components of innate antifungal defense, yet the mechanisms by which they detect fungal morphology have remained unclear. We show that NKp46 (NCR1 in mice), an activating receptor on NK cells, directly recognizes the hyphal form of C. albicans but not yeast. Using image flow cytometry and receptor–ligand binding assays with NKp46-Ig and NCR1-Ig fusion proteins, we demonstrate that NKp46 engagement is mediated through its D2 domain and is independent of sialic acid interactions. Functional blockade of NKp46 significantly reduces NK cell degranulation and fungal killing, while NCR1-deficient mice display heightened susceptibility to systemic candidiasis. These findings identify NKp46 as a critical sensor of fungal morphology and highlight its importance in shaping early antifungal immunity.

## Highlights

• *Ncr1* knockout mice lose resistance to *Candida albicans* infection
• NCR1/NKp46 binds *C. albicans* hyphae but not yeast cells
• NKp46-specific antibody 461-G1 inhibits the NK cell–mediated killing of *C. albicans*

## Graphical abstract

**Figure:**
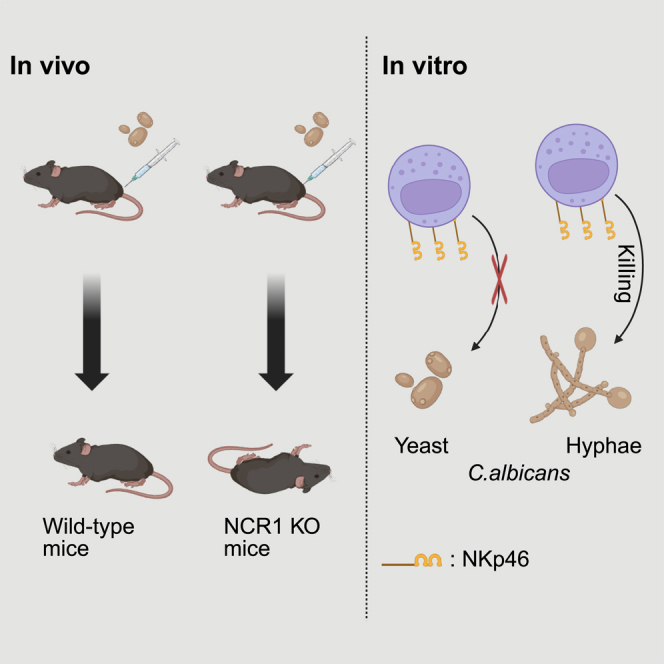

## Introduction

*Candida albicans* is a commensal fungus that asymptomatically colonizes human mucosal surfaces such as the skin, oral cavity, gastrointestinal tract, and urogenital tract.^1^ However, under conditions of immune suppression or antibiotic use, *C. albicans* can transition from a benign commensal to a pathogenic invader, leading to mucosal infections or life-threatening systemic candidiasis.^2^ Despite antifungal therapy, mortality from systemic *C. albicans* infections often exceeds 40%.^3^^,^^4^

One of the major virulence traits of *C. albicans* is its ability to undergo morphological transitions between yeast, pseudohyphal, and true hyphal forms.^2^ The yeast form is associated with colonization and dissemination, whereas the hyphal form exhibits enhanced tissue penetration and immune evasion.^3^ Recognition and clearance of the hyphal form are thus essential components of effective antifungal immunity.^5^^,^^6^

Natural killer (NK) cells are innate lymphocytes that contribute to host defense against tumors, viruses, and fungi.^7^^,^^8^^,^^9^^,^^10^^,^^11^ Their cytotoxic activity is regulated by a balance of activating and inhibitory receptors.^12^ In fungal immunity, NK cells have been shown to utilize receptors such as TIGIT and NKG2D to recognize *C. albicans*, while NKp46 is implicated in the recognition of *C. glabrata*, but not *C. albicans*.^7^^,^^13^^,^^14^

NKp46 (NCR1 in mice) is a natural cytotoxicity receptor that mediates the activation of NK cells in response to a wide array of pathogens.^7^^,^^15^ The amino acid sequences of NKp46 and NCR1 share 58% identity.^16^ Both receptors possess two extracellular immunoglobulin-like domains (D1 and D2), with the D2 domain serving as the primary mediator of ligand binding.^17^ NCR1 contains 3 N-glycosylation sites, NKp46 contains 2 O-glycosylation sites and 1 N-glycosylation site.^18^ Here, we provide evidence that NKp46 does bind *C. albicans*—specifically in its hyphal form—thus redefining the role of NKp46 in fungal immunity.

## Results

### NCR1-deficient mice exhibit increased susceptibility to systemic C. albicans infection

**Figure 1:**
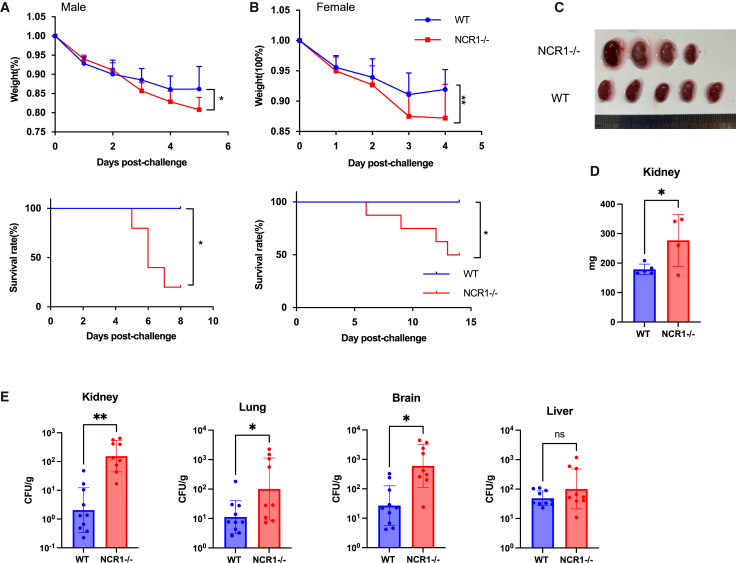
NCR1 knockout (KO) mice exhibit reduced resistance to *C. albicans* infection (A) Wild-type (WT) (*n* = 5) and NCR1 KO (*n* = 5) male mice were injected intravenously with *C. albicans* (0.25M). Body weight was recorded daily. Survival rates of *C. albicans*-infected WT and NCR1 KO mice were assessed. Body weight and Survival curves were generated from the pooled data of two independent experiments. Data are represented as mean ± SD. Statistical significance for weight differences was analyzed using two-way ANOVA with Šídák’s multiple comparisons test. Survival curves were analyzed using the Kaplan-Meier method, and statistical significance was determined by the Log rank (Mantel-Cox) test. Statistical significance is indicated as follows: *p* < 0.05 (∗), *p* < 0.01 (∗∗). (B) Wild-type (WT) (*n* = 10) and NCR1 KO (*n* = 8) female mice were injected intravenously with *C. albicans* (0.25M). Body weight was recorded daily. Survival rates of *C. albicans*-infected WT and NCR1 KO mice were assessed. Body weight and Survival curves were generated from the pooled data of two independent experiments. Data are represented as mean ± SD. Statistical significance for weight differences was analyzed using two-way ANOVA with Šídák’s multiple comparisons test. Survival curves were analyzed using the Kaplan-Meier method, and statistical significance was determined by the Log rank (Mantel-Cox) test. Statistical significance is indicated as follows: *p* < 0.05 (∗), *p* < 0.01 (∗∗). (C) Gross pathology of kidneys 2 days post-infection from WT (*n* = 5) and NCR1 KO (*n* = 4) mice. (D) Kidney mass from WT and NCR1 KO mice was measured 2 days post-infection. Each dot represents an individual mouse. Data are represented as mean ± SD. Statistical significance was determined using an unpaired two-tailed Student’s t test. Statistical significance is indicated as follows: *p* < 0.05 (∗), *p* < 0.01 (∗∗). (E) Fungal burden in the kidney, lung, brain, and liver 2 days post *C. albicans* infection. Organs were homogenized and serially diluted (10×, 100×, 1000×). Diluted samples were plated on Sabouraud agar and incubated overnight at 30°C. Colonies were counted, and fungal burden was quantified. Each dot represents an individual mouse. Data are represented as mean ± SD. Experiments were repeated twice. Statistical significance was determined using an unpaired two-tailed Student’s t test (*p* < 0.05 was considered statistically significant). Statistical significance is indicated as follows: *p* < 0.05 (∗), *p* < 0.01 (∗∗).

To determine the *in vivo* relevance of NKp46 in host defense against *C. albicans*, we intravenously injected male and female wild-type and NCR1 knockout mice with *C. albicans* and monitored survival and body weight. NCR1-deficient mice displayed significantly increased mortality and more rapid weight loss compared to wild-type controls, in both male and female mice (Figures 1A and 1B).

At day 2 post-infection, gross examination revealed enlarged kidneys in NCR1-deficient mice (Figure 1C), with significantly increased kidney mass compared to wild-type mice (Figure 1D). Fungal burden was also significantly elevated in multiple organs, including the kidney, brain, and lung (Figure 1E), suggesting compromised fungal clearance in the absence of NCR1.

We also examined the cell frequencies of CD4^+^ T cells, CD8^+^ T cells, NK cells, and neutrophils in mouse spleens before and after *C. albicans* injection. NCR1^−/−^ mice exhibited reduced numbers of CD4^+^ and CD8^+^ T cells, while the numbers of NK cells and neutrophils remained largely unchanged (Figure S1). These results imply that NK cells may mediate antifungal immunity against *C. albicans* not only through direct cytotoxic mechanisms but also indirectly by shaping the CD4^+^ and CD8^+^ T cell compartments.

NK cells have been reported to play an important role in *C. albicans* skin infection models involving deep dermal injections, where NK cell-depleted mice exhibit reduced ulceration compared to wild-type controls.^19^ In their model, mice were injected with *C. albicans* hyphae into the deep dermis, and lesion progression was assessed over time. In wild-type (WT) mice, skin ulceration typically developed around 2 days post-infection. In contrast, ulceration was less frequent among NK cell–depleted mice. Instead, these mice often developed encapsulated abscesses containing *C. albicans* without overt ulcer formation. Based on these findings, we also investigated whether NCR1 contributes to this process. In contrast, the incidence of ulceration did not differ significantly between NCR1^−/−^ mice and WT animals (Figure S2), indicating that NKp46 plays a context-dependent role in host defense, particularly in systemic candidiasis.

### NKp46-Ig and NCR1-Ig specifically recognize the hyphal form of C. albicans

**Figure 2:**
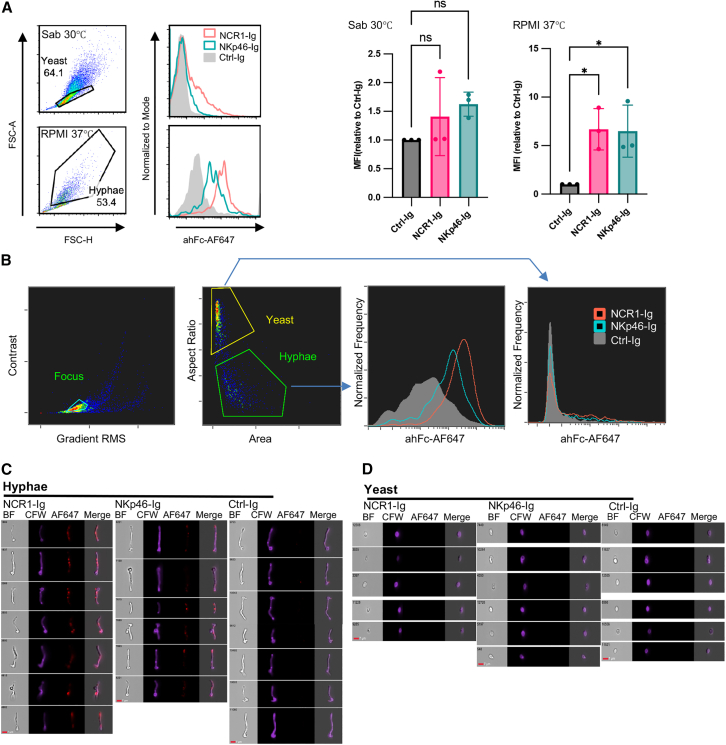
NKp46-Ig and NCR1-Ig specifically bind to the hyphal form of *C. albicans* (A) *C. albicans* was cultured in Sabouraud medium at 30°C to maintain the yeast form (upper left) or in RPMI at 37°C (lower left) to induce hyphal formation. Cells were stained with NKp46-Ig or NCR1-Ig (right histograms), followed by an anti-human Fc Alexa Fluor 647 (AF647)-conjugated secondary antibody. Experiments were independently performed three times, and the MFI values from each experiment were pooled for statistical analysis. Data are represented as mean ± SD. Statistical analysis was performed using ordinary one-way ANOVA followed by Tukey’s multiple comparisons test. Statistical significance is indicated as follows: *p* < 0.05 (∗). (B) Gating strategy for image flow cytometry analysis of *C. albicans* cultured in RPMI at 37°C. Cells were stained with Calcofluor White (CFW) and NKp46-Ig or NCR1-Ig, followed by ahFc-AF647. Focused cells were initially gated based on “Contrast” and “Gradient RMS.” Yeast and hyphal forms were then distinguished using “Aspect Ratio” and “Area.” (C) Representative images of *C. albicans* in the hyphal form from (B). Brightfield (BF), CFW staining, fusion protein staining, and the merged channel are shown. (D) Representative images of *C. albicans* in the yeast form from (B). Brightfield (BF), CFW staining, fusion protein staining, and the merged channel are shown. All scale bars, 7 μm.

The *in vivo* findings suggest that *C. albicans* may express a ligand recognized by NCR1. To investigate the potential recognition of *C. albicans* by NKp46, we stained *C. albicans* in different morphological states. *C. albicans* cultured at 30°C in Sabouraud medium remained in the yeast form, while incubation in RPMI at 37°C induced hyphal formation. Cells were stained with NKp46-Ig or NCR1-Ig and analyzed by flow cytometry. Gating based on forward and side scatter parameters allowed the separation of yeast and hyphal populations (Figure 2A, left). After 3 h incubation in RPMI, approximately 50% of *C. albicans* formed hyphae. We observed strong binding of both NKp46-Ig and NCR1-Ig to the hyphal form, while no significant binding was detected on the yeast form (Figure 2A, right).

To determine whether NKp46 binding is driven by morphological changes or culture conditions, we further analyzed C. albicans cultured in RPMI at 37°C for 3 hours using imaging flow cytometry. Morphological distinction between yeast and hyphae was achieved by gating focused cells based on “Aspect Ratio” and “Area” parameters (Figure 2B). Tightly gated populations of *C. albicans* yeast and hyphal forms were compared by immunofluorescence intensity to evaluate the differential binding of fusion proteins to the two fungal morphologies. We observed that significant binding of NCR1-Ig and NKp46-Ig occurred only in the hyphal form, whereas minimal or no binding was detected in the yeast form.

To further validate the accuracy of yeast and hyphae gating, representative images of stained hyphal and yeast form cells were presented (Figures 2C and 2D). Consistent with our previous results, NKp46-Ig and NCR1-Ig bound almost exclusively to hyphal cells. The binding of NKp46-Ig and NCR1-Ig was not restricted to the elongated hyphal segments but was also observed on the yeast-like regions of the fungal cells. These findings indicate that the morphological switch to hyphae, rather than the culture environment itself, determines NKp46 ligand expression. Labeled *C. albicans* cells were also examined under a fluorescence microscope to rule out potential artifacts or effects caused by flow cytometry (Figure S3).

### Structural requirements and binding specificity of NKp46 to C. albicans

**Figure 3:**
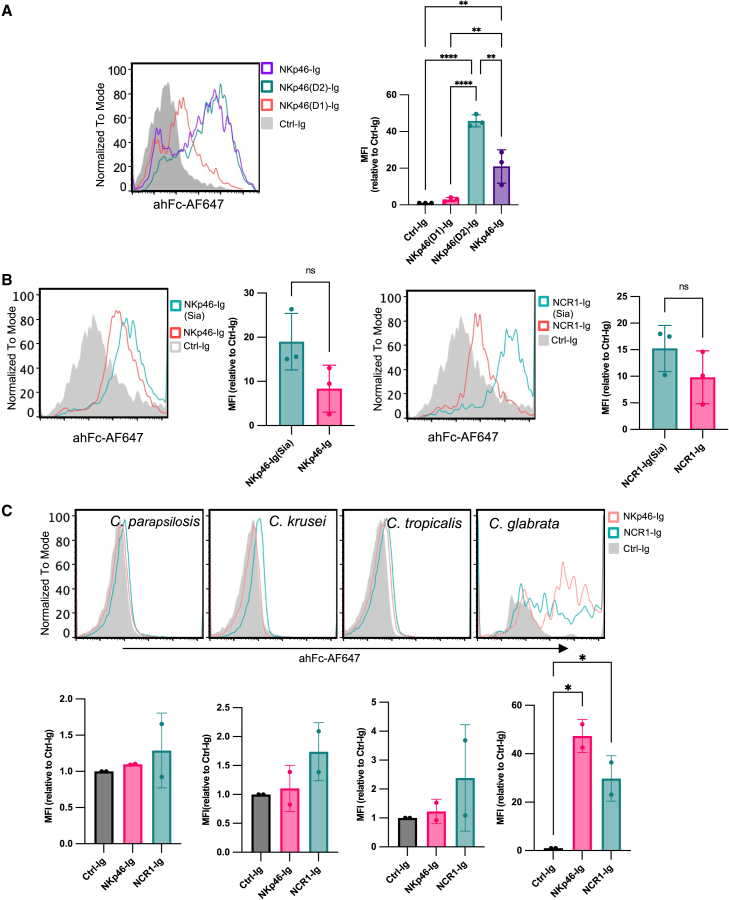
Binding properties of NKp46 to *C. albicans* (A) Full-length NKp46-Ig and its individual D1-Ig and D2-Ig domains were used to stain hyphal *C. albicans*, followed by flow cytometry (FACS) analysis. Experiments were independently performed three times, and the MFI values from each experiment were pooled for statistical analysis. Data are represented as mean ± SD. Statistical analysis was performed using ordinary one-way ANOVA followed by Tukey’s multiple comparisons test. Statistical significance is indicated as follows: *p* < 0.05 (∗). (B) Hyphal *C. albicans* was stained with NKp46-Ig (left) and NCR1-Ig (right), with or without prior sialidase (Sia) treatment, followed by FACS analysis. Experiments were independently performed three times, and the MFI values from each experiment were pooled for statistical analysis. Data are represented as mean ± SD. Statistical significance was determined using an unpaired two-tailed Student’s t test (*p* < 0.05 was considered statistically significant). (C) *C. parapsilosis*, *C. krusei*, *C. tropicalis*, and *C. glabrata* were cultured in RPMI at 37°C, then stained with NKp46-Ig and NCR1-Ig, followed by an anti-human Fc AF647-conjugated secondary antibody. Experiments were independently performed twice, and the MFI values from each experiment were pooled for statistical analysis. Data are represented as mean ± SD. Statistical analysis was performed using ordinary one-way ANOVA followed by Tukey’s multiple comparisons test. Statistical significance is indicated as follows: *p* < 0.05 (∗).

To dissect the structural basis of NKp46 binding to hyphal *C. albicans*, we used fusion proteins containing individual NKp46 domains (D1-Ig and D2-Ig). Flow cytometry revealed that the D2 domain alone was sufficient to mediate binding to hyphal *C. albicans*, indicating it plays a critical role in ligand recognition (Figure 3A).

Given the importance of sialic acid in other NKp46-ligand interactions,^17^^,^^20^ we treated NKp46-Ig and NCR1-Ig with sialidase prior to staining. Unexpectedly, sialidase treatment enhanced rather than diminished the binding of both NKp46-Ig (Figure 3B, left) and NCR1-Ig to *C. albicans* hyphae (Figure 3B, right), suggesting that sialic acid on NKp46 may partially mask its binding capacity in this context.

We next evaluated NKp46 and NCR1 binding to other *Candida* species under hyphae-inducing conditions of *C. albicans*, although only *C. parapsilosis* has been reported to form true hyphae among them.^21^ Consistent with previous findings, NKp46-Ig and NCR1-Ig bound to *C. glabrata*, but not to other species.^7^

To try to identify the hyphal ligand of NKp46, we stained several *C. albicans* mutants lacking major adhesins and observed no change in NKp46-Ig binding, suggesting that NKp46 does not recognize known surface adhesins (Figure S4). To further investigate potential ligands, we assessed whether TIGIT-Ig and NKG2D-Ig influence NKp46/NCR1-Ig binding to *C. albicans*. The results indicated no impact, suggesting that these receptors recognize distinct ligands without cross-interference (Figure S5).

### Blocking NKp46 impairs NK cell-mediated responses to C. albicans

**Figure 4:**
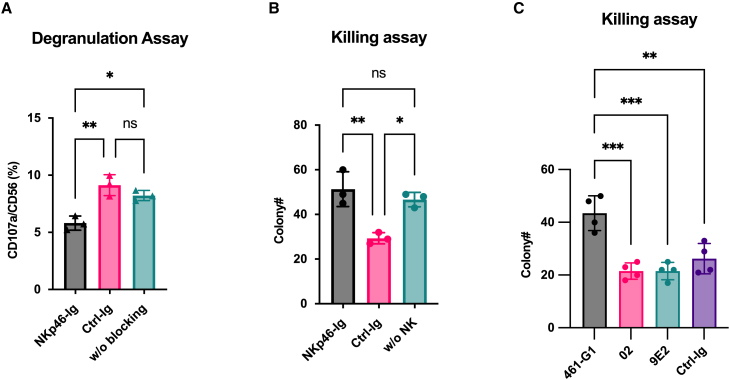
Blocking NKp46 reduces human NK cell-mediated killing of *C. albicans* (A) Human NK cells were co-cultured with *C. albicans* for 4 h with or without the indicated Ig proteins (X axis) and stained with anti-CD56-PE and anti-CD107a-APC antibodies. The percentage of CD107a^+^ expression on CD56^+^ NK cells was analyzed by flow cytometry. Each dot denotes a single replicate in the experiment. Data are represented as mean ± SD. Experiments were repeated twice. Statistical analysis was performed using ordinary one-way ANOVA followed by Tukey’s multiple comparisons test. Statistical significance is indicated as follows: *p* < 0.05 (∗), *p* < 0.01 (∗∗). (B) *C. albicans* and human NK cells were co-cultured overnight with the indicated fusion proteins, and the number of viable *C. albicans* cells was quantified by serial dilution and plating on agar. Each dot denotes a single replicate in the experiment. Data are represented as mean ± SD. Experiments were repeated twice. Statistical analysis was performed using ordinary one-way ANOVA followed by Tukey’s multiple comparisons test. Statistical significance is indicated as follows: *p* < 0.05 (∗), *p* < 0.01 (∗∗). (C) *C. albicans* and human NK cells were co-cultured overnight with indicated clones of anti-NKp46 antibodies, and the number of viable *C. albicans* cells was quantified by serial dilution and plating on agar. Each dot denotes a single replicate in the experiment. Data are represented as mean ± SD Experiments were repeated twice. Statistical analysis was performed using ordinary one-way ANOVA followed by Tukey’s multiple comparisons test. Statistical significance is indicated as follows: *p* < 0.01 (∗∗), *p* < 0.001 (∗∗∗), and *p* < 0.0001 (∗∗∗∗).

To assess the functional relevance of NKp46 binding to hyphal *C. albicans*, we performed NK cell degranulation and killing assays. For this we used the NKp46-Ig protein as a competitive inhibitor. Human NK cells were co-cultured with *C. albicans* in the presence or absence of NKp46-Ig fusion protein. The addition of NKp46-Ig significantly reduced CD107a expression on NK cells (Figure 4A) and impaired their ability to kill *C. albicans* (Figure 4B), indicating that the NKp46-mediated recognition of hyphae contributes to effective antifungal activity.

Surprisingly, when we attempted to use different clones of anti-NKp46 antibodies to block the interactions between NKp46 and *C. albicans*, we found that clone 461-G1 exhibited significant blocking activity, whereas clones 02 and 9E2 did not (Figure 4C). Although 461-G1 targets the D1 domain of NKp46, it is possible that 461-G1 induces steric hindrance on NKp46, thereby preventing *C. albicans* from further binding to the D2 domain.^17^

## Discussion

Natural killer (NK) cells play a critical role in antifungal immunity, including against *C. albicans* and other pathogenic fungi^7^^,^^13^^,^^14^ Several NK cell receptors—such as TIGIT and NKG2D—have been shown to modulate NK cell responses to *C. albicans*.

The ability to distinguish between yeast and hyphal forms of *C. albicans* is crucial for the immune system. Hyphae are considered the more invasive morphology and are essential for mucosal penetration.^4^^,^^19^ It has been previously shown that some immune molecules, such as Dectin-2 and mannose-binding lectin (MBL), preferentially bind the hyphal form of *C. albicans*, potentially enabling the immune system to target the most pathogenic form of the fungus.^22^^,^^23^ Neutrophils, for example, exhibit enhanced phagocytosis and killing of hyphae compared to yeast, suggesting that the early recognition of hyphae is a critical component of the antifungal response.^24^ Moreover, since the hyphal form of *C. albicans*—as opposed to the yeast form—is primarily responsible for epithelial invasion and pathogenicity, the selective recognition of hyphae by NKp46 and the pattern recognition receptor Dectin-2 renders NK cell-mediated killing more targeted. This specificity facilitates a rapid immune response during the early stages of infection, thereby preventing further dissemination. Even after infection is established, the continued recognition of hyphae by immune cells may help minimize tissue damage and mitigate the pathogenic potential of *C. albicans*.

Our data show that NKp46 is another pattern recognition receptor capable of selectively identifying *C. albicans* hyphae. This interaction is mediated through the D2 domain of NKp46 and is independent of sialic acid, which is commonly involved in ligand interactions with NKp46 in other contexts, such as viral and tumor recognition.^17^ Interestingly, removal of sialic acid from NKp46 increased its binding to hyphae, suggesting a possible steric hindrance by sialic acid residues. These findings raise the possibility that NKp46 recognizes a non-sialylated fungal ligand, potentially a carbohydrate or glycoprotein component of the hyphal cell wall.

Functionally, NKp46 binding to *C. albicans* hyphae is important for effective NK cell activation, as blocking this interaction significantly impairs NK cell degranulation and fungal killing. *In vivo*, NCR1-deficient mice were more susceptible to systemic *C. albicans* infection, exhibiting greater mortality, increased fungal burden, and severe kidney pathology. These data underscore the importance of NKp46 in mediating early immune responses against disseminated candidiasis.

Although the precise ligand of NKp46 on *C. albicans* remains to be identified, our attempts to screen knockout strains for loss of binding and to immunoprecipitate potential ligands from the fungal cell wall were inconclusive. This could be due to the fact that many knockout strains affecting hyphal formation also alter cell wall architecture, confounding the identification of specific ligands.^25^^,^^26^ Nevertheless, the binding pattern and biochemical characteristics of this interaction suggest that the NKp46 ligand may be a hyphae-specific polysaccharide or an uncharacterized glycoprotein exposed during morphogenesis.

Our findings offer a novel therapeutic avenue for combating *C. albicans* infection, for example, by enhancing the expression of NKp46 on NK cells through specific approaches. Given that certain patients with cancer are co-infected with *C. albicans*, we propose that CAR-NK cells engineered to overexpress NKp46 could potentially serve a dual purpose—targeting both fungal infection and tumor cells simultaneously.

In summary, our study demonstrates that NKp46 is a functional receptor for *C. albicans* hyphae, contributing to NK cell-mediated antifungal immunity. This finding not only expands our understanding of NK cell recognition mechanisms in fungal infection but also highlights the importance of immune discrimination between commensal and pathogenic fungal forms. Targeting NKp46-ligand interactions may offer new strategies for enhancing antifungal immunity, particularly in immunocompromised patients at risk for invasive candidiasis.

### Limitations of the study

Although this study establishes a protective role of the NCR1 receptor against *C. albicans* infection in mice, the contributions of other immune cell populations within this model, as well as their crosstalk with NK cells, remain incompletely understood. Future studies should quantify cytokine profiles in NCR1-deficient mice and clarify how NK cells influence the functional behavior of other immune subsets. Moreover, elucidating these ligands is essential to unraveling the mechanisms of NK cell function and host defense against *C. albicans*. Despite efforts to identify ligands for NKp46/NCR1, this remains unresolved, underscoring the need for alternative strategies in subsequent research.

## Resource availability

### Lead contact

Further information and requests for resources should be directed to and will be fulfilled by the Lead contact, Ofer Mandelboim at oferm@ekmd.huji.ac.il.

### Materials availability

Newly generated materials reported in this article are available from the lead contact upon request.

### Data and code availability

• All analyzed and raw data reported in this article will be shared by the lead contact upon request and has not been deposited online due to patent filing proceedings.
• This article does not report original code.
• Any additional information required to reanalyze the data reported in this article is available from the lead contact upon request.

## Acknowledgments

The authors would like to thank Prof. Brendan Cormack for supplying critical fungal strains. We would like to also thank the Fungal Genomic Stock Center (FGSC) for providing the *C. albicans* deletion library.^27^ Ofer Mandelboim is supported by: The Israel Science Foundation grant 3042/22, grant 442/18, and grant 619/23. The Israel Innovation Authority grant 75934. The ICRF professorship grant. The ISF Israel-China grant 2554/18.

## Author contributions

Mingdong Liu, Ofer Mandelboim. Funding acquisition: Ofer Mandelboim. Investigation: Mingdong Liu, Ahmed Rishiq, Yoav Charpak-Amikam, Fubin Li. Methodology: Mingdong Liu, Ahmed Rishiq, Yoav Charpak-Amikam. Supervision: Ofer Mandelboim. Writing – original draft: Mingdong Liu. Writing – review and editing: Ofer Mandelboim.

## Declaration of interests

The authors declare no competing interests.

## STAR★Methods

### Key resources table

**Table:** 

REAGENT or RESOURCE	SOURCE	IDENTIFIER
**Antibodies**		

Anti human CD56 antibody	BioLegend	Cat# 318304; RRID: AB_604100
Anti human CD107a antibody	BioLegend	Cat# 328620; RRID: AB_1279055
Anti human Fc antibody	JacksonImmunoResearch	Cat# 709-606-098; RRID: AB_2340580
Anti mouse CD45 antibody	BioLegend	Cat# 103149; RRID: AB_2564590
Anti mouse CD4 antibody	BioLegend	Cat# 100429; RRID: AB_493698
Anti mouse CD8 antibody	BioLegend	Cat# 100712; RRID: AB_312751
Anti mouse NK1.1 antibody	BioLegend	Cat# 156503; RRID: AB_2783135
Anti mouse Ly6G antibody	BioLegend	Cat# 127608; RRID: AB_1186099
Anti mouse CD11b antibody	BioLegend	Cat# 982604; RRID: AB_2632619
Anti mouse CD3 antibody	BioLegend	Cat# 317343; RRID: AB_2565848
Anti-NKp46	BioLegend	Cat# 331918; RRID: AB_2561650
Anti-NKp46(02)	Berhani et al.^28^	N/A
Anti-NKp46(461-G1)	Arnon et al.^17^	N/A
Anti-NCR1	BioLegend	Cat# 137645; RRID: AB_2876479

**Chemicals, peptides, and recombinant proteins**		

CFW	BioTrend	Cat# 29067
DAPI	ThermoFisher	Cat# TS-62248
Recombinant human IL-2	PeproTech	Cat#200-02-1000
Neuraminidase	Sigma	Cat#N-5254

**Experimental models: Organisms/strains**		

Ncr1^*gfp/gfp*^ knockout (KO) mice	In House	B6;129-Ncr1tm1Oman/J; RRID: IMSR_JAX:022739
*Candida albicans* SC5314	In House	N/A
*Candida glabrata* BG2	In House	N/A
*Candida parapsilosi*	In House	N/A
*Candida krusei*	In House	N/A
*C. albicans* deletion mutants *als*1Δ/Δ1467, als2Δ/Δ 2757, als3Δ/Δ 1843, als4Δ/Δ 2034, als5Δ/Δ 2373, als6Δ/Δ 1420, als7Δ/Δ 1429 and als9Δ/Δ 2028	In House	N/A
C. albicans deletion mutants *ECE1Δ/Δ, HWP2Δ/Δ, HYR1Δ/Δ, RBT1Δ/Δ*	Fungal Genomic Stock Center (FGSC)	Knockout set from Suzanne Noble

**Critical commercial assays**		

EasySep™ Human NK Cell Enrichment Kit	STEMCELL Technologies	Cat#17955

**Software and algorithms**		

Prism9	GraphPad Software	https://www.graphpad.com/scientific-software/prism/
FlowJo10	BD Research	https://www.flowjo.com/
Fiji	Schneider et al.^29^	https://imagej.net/software/fiji
IDEAS® Software	amins	Version 6.0

### Experimental model and study participant details

#### Mice

Both male and female Ncr1^gfp/gfp^ knockout (KO) mice (RRID: IMSR_JAX:022739) and C57BL/6 mice (6–8 weeks old; Envigo, Israel) were used in this study. All animals were specific pathogen-free (SPF), experimentally naïve, and group-housed. Littermates were randomly assigned to experimental groups. All procedures were performed in the SPF animal facility of the Hebrew University-Hadassah Medical School (Ein Kerem, Jerusalem), following the Declaration of Helsinki and institutional ethical guidelines. The experiment was approved by the Ethics Committee of the Hebrew University of Jerusalem under the approval number: MD-22-16975-5.

#### Fungal strains

The fungal strains used included *Candida albicans* SC5314, *Candida glabrata*, *Candida parapsilosis*, *Candida krusei*, and a panel of *C. albicans* deletion mutants (*als1*–*als7*Δ/Δ, *als9*Δ/Δ, *ECE1*Δ/Δ, *HWP2*Δ/Δ, *HYR1*Δ/Δ, *RBT1*Δ/Δ), all derived from SC5314.

Fungi were stored at −80°C in glycerol stocks and streaked onto Sabouraud Dextrose Agar (SDA; Sigma) for up to 4 weeks. For experiments, colonies were cultured overnight in Sabouraud broth at 30°C with shaking, diluted 1:50 in fresh broth, and grown for an additional 2–4 h. For hyphal induction, cells were further diluted 1:20 into RPMI-1640 medium and incubated as above.

#### Human NK cell

Human NK cells were isolated from peripheral blood mononuclear cells (PBMCs) of healthy male or female donors using the EasySep™ Human NK Cell Enrichment Kit (STEMCELL Technologies). NK cells (5 × 10^4^/well) were co-cultured in U-bottom 96-well plates with irradiated (6000 RAD) PBMCs from two independent donors (5 × 10^4^/well per donor) and RPMI-8866 cells (5 × 10^3^/well). Cells were maintained in RPMI 1640 supplemented with 10% human serum (Sigma), 1 mM sodium pyruvate, 2 mM glutamine, non-essential amino acids, 100 U/mL penicillin, 0.1 mg/mL streptomycin (all from Biological Industries), 500 U/mL recombinant human IL-2 (PeproTech), and 20 μg/mL phytohemagglutinin (PHA; Sigma). Cultures were maintained at 37°C in 5% CO_2_. NK cell identity was verified by flow cytometry using anti-human CD56-PE and CD3-FITC antibodies (BioLegend).

### Method details

#### Mouse infection

For systemic infection model, on the day of infection, C. albicans cells were washed 3 times in PBS and kept on ice. Mice were intravenously injected with 2.5 × 10^5^ CFU in 100 μL PBS via the tail vein. At 48 h post-infection, mice were euthanized and organs harvested. Organs were homogenized, filtered through a 70 μm strainer, serially diluted in PBS, and plated on SDA. CFUs were counted after 48 h of incubation at 30°C. Mice were monitored daily. Animals were euthanized if body weight dropped below 80% of baseline or if severe clinical signs developed.

For skin infection model, at least 24 hours before injection, mice had their dorsal region shaved. Subsequently, they were injected intradermally with either C. albicans hyphae (5 × 10^6^ or 20 × 10^6^ in a total volume of 50 μl) at the shaved dorsal site. Skin ulceration was then assessed macroscopically at 1, 2, and 3 days post-injection.

#### Flow cytometry and imaging flow cytometry

Mammalian and fungal cells were prepared as described above, washed 3 times in ice-cold PBS (515 g for mammalian, 3000 g for fungi, 5 min at 4°C), and seeded into U-bottom 96-well plates (5–10 × 10^4^ cells/well). For blocking, cells were incubated with 2.5 μg/well of antibody for 30 min on ice. Cells were stained with 0.25 μg/well primary antibodies or 0.5–5 μg/well fusion proteins in FACS buffer (PBS, 0.05% BSA, 0.05% NaN_3_) for 1 h on ice. If necessary, cells were then stained with 0.75 μg/well fluorophore-conjugated secondary antibodies (AlexaFluor647 or APC; Jackson ImmunoResearch) for 30–45 min. Data were acquired on a CytoFlex (Beckman-Coulter) or ImageStream®X Mark II and analyzed using FlowJo software and IDEAS® Software.

#### Fusion proteins

The extracellular domains of the fusion proteins were cloned into a mammalian expression vector (pIRESpuro3) containing a mutated human IgG1 Fc domain. The resulting Ig-expression vectors were transfected into 293T cells (CRL-3216, ATCC), which were subsequently cultured in the continuous presence of puromycin. Fusion proteins secreted into the culture medium were purified using a HiTrap Protein G High Performance column (Cat# GE17-0405-01, GE Healthcare). Fusion proteins included NKp46-Ig, NKp46-D1-Ig, NKp46-D2-Ig, NTBA-Ig (negative control), TIGIT-Ig, NKG2D-Ig and mouse NCR1-Ig.

Desialylation of NKp46-Ig was performed by incubation with 0.015 U insoluble neuraminidase (NA) conjugated to beaded agarose (N-5254; Sigma, St. Louis, MO) or with PBS (control) for 1.5 h at 17 °C on a roller.

#### Fungal killing and degranulation assays

Effector cells were blocked with 02, 9E2, 461-G1, NTBA-Ig or anti-NKp46-Ig (10 μg/well) for 1 h on ice. Cells were co-cultured with fungal targets (5 × 10^4^ effector cells and 1 × 10^3^ fungi/well) in RPMI-1640 medium for 12–14 h at 37°C in 5% CO_2_. After incubation, cultures were serially diluted in PBS and plated on SDA. Plates were incubated at 30°C for 24–48 h, and colony-forming units (CFUs) were counted.

For degranulation assays, NK cells were co-cultured with fungal cells as described above. Anti-CD56-PE and anti-CD107a-APC antibodies (0.5 μl each per well) were added to the culture at the beginning of co-incubation. After 2 hours, cells were washed twice with cold FACS buffer and analyzed using a CytoFlex flow cytometer (Beckman-Coulter).

#### Graphic illustrations

The graphic illustrations displayed in this paper were created with BioRender.com.

### Quantification and statistical analysis

Two-way ANOVA with Šídák’s multiple comparisons test, ordinary one-way ANOVA followed by Tukey’s multiple comparisons test, survival analysis and student’s T-tests in GraphPad Prism version 9 were conducted, and the statistical significance, value of n, meaning of n and definition of center and dispersion are indicated in the figure legends. Data in the figures are represented as mean ± SD. *p* < 0.05 (∗), *p* < 0.01 (∗∗), *p* < 0.001 (∗∗∗), *p* < 0.0001 (∗∗∗∗).
