# Supplementary material for: NKp46 recognizes the hyphal form of Candida albicans and mediates protective antifungal immunity
Source: iScience. 2025 Sep 12;28(10):113556. doi: 10.1016/j.isci.2025.113556 (PMC12513223; doi:10.1016/j.isci.2025.113556)
Supplement: Document S1. Figures S1–S5 [file mmc1.pdf]

iScience, Volume *28*

Supplemental information

NKp46 recognizes the hyphal form of Candida albicans and mediates protective antifungal immunity

Mingdong Liu, Ahmed Rishiq, Yoav Charpak-Amikam, Fubin Li, and Ofer Mandelboim

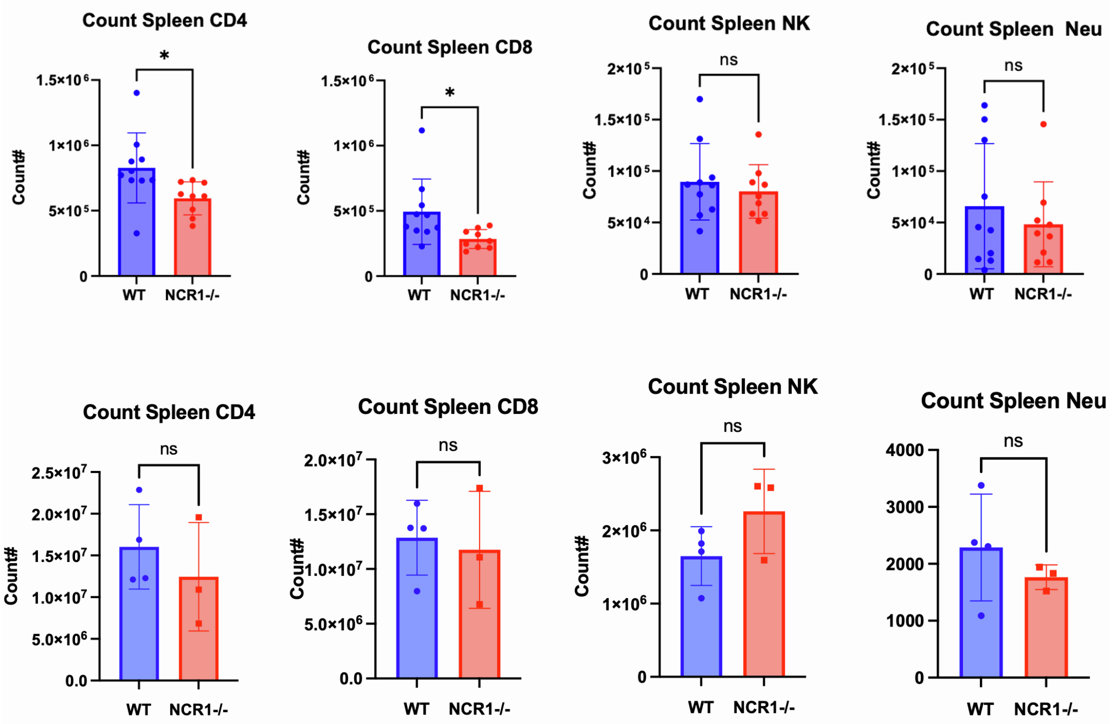

Supplementary Figure 1. NCR1-KO mice exhibit a decreased cell number of CD4 and CD8 T cells in the spleen compared to wild-type controls. Related to Figure 1.

(A) Absolute numbers of $CD4^+$ T cells, $CD8^+$ T cells, NK cells, and neutrophils in the spleen of uninfected NCR1 KO (n=10) and WT (n=9) mice were analyzed by flow cytometry. (B) Absolute numbers of CD4 T cells, CD8 T cells, NK cells and neutrophils in the spleen of NCR1 KO (n=3) and WT (n=4) mice after C. albicans injection were analyzed by flow cytometry. Each dot represents an individual mouse. Data are represented as mean +/- SD. Statistical significance was determined using an unpaired two-tailed Student's t-test ($P < 0.05$ was considered statistically significant). Statistical significance is indicated as follows: $P < 0.05$ (*), $P < 0.01$ (**).

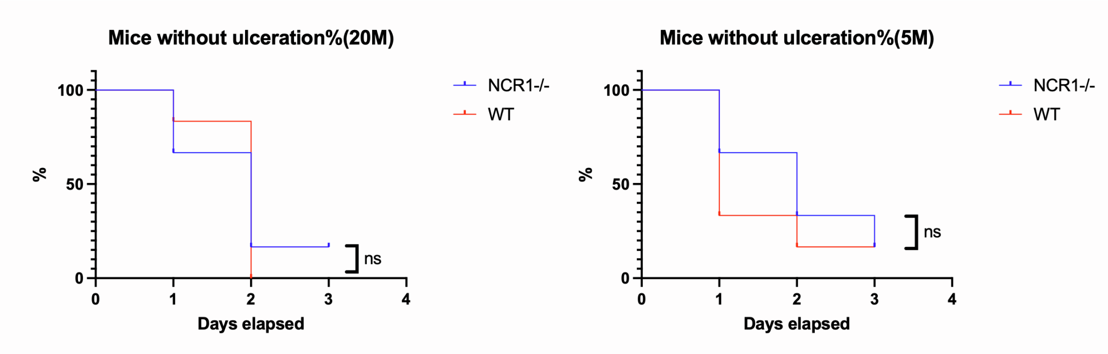

Supplementary Figure 2. No significant difference between WT and NCR1 KO mice in a skin infection model. Related to Figure 1.

Percentage of WT(n=6) and NCR1 KO (n=6) mice developing ulceration following *C. albicans* administration. Ulceration curves were generated from pooled data of two independent experiments. Data are represented as mean +/- SD. Survival analysis was performed using the Kaplan-Meier method, and statistical significance was determined by the Log-rank (Mantel-Cox) test. Statistical significance is indicated as follows: $P < 0.05$ (*), $P < 0.01$ (**).

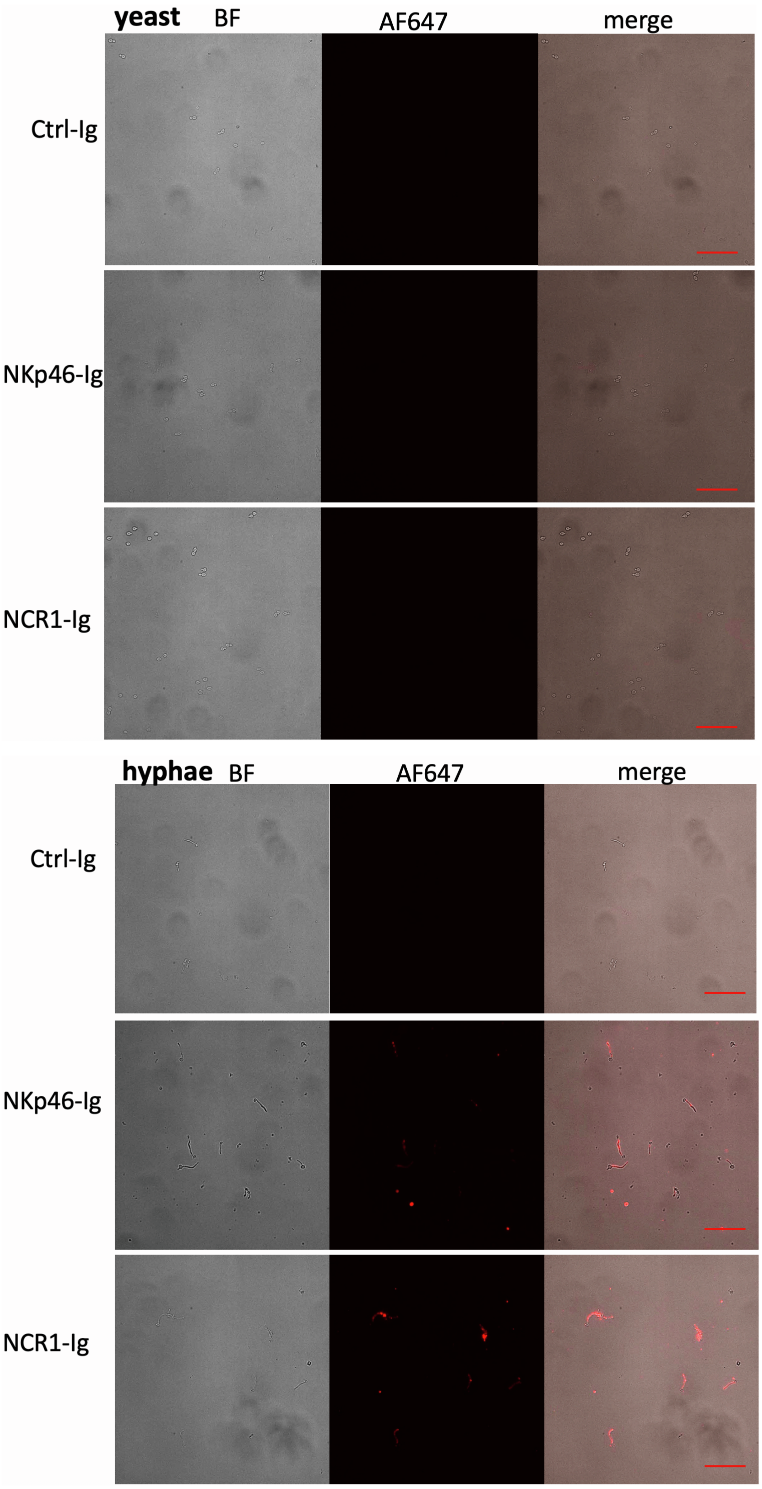

Supplementary Figure 3. NKp46-Ig and NCR1-Ig bind to hyphae of *C. albicans*. Related to Figure 2.

Yeast and hyphal forms of *C. albicans* were incubated with recombinant NKp46-Fc or NCR1-Fc fusion proteins, followed by staining with Alexa Fluor 647-conjugated anti-human Fc secondary antibody. Samples were imaged under a fluorescence microscope at 40× magnification. Representative images are shown. All scale bars, 60 μm.

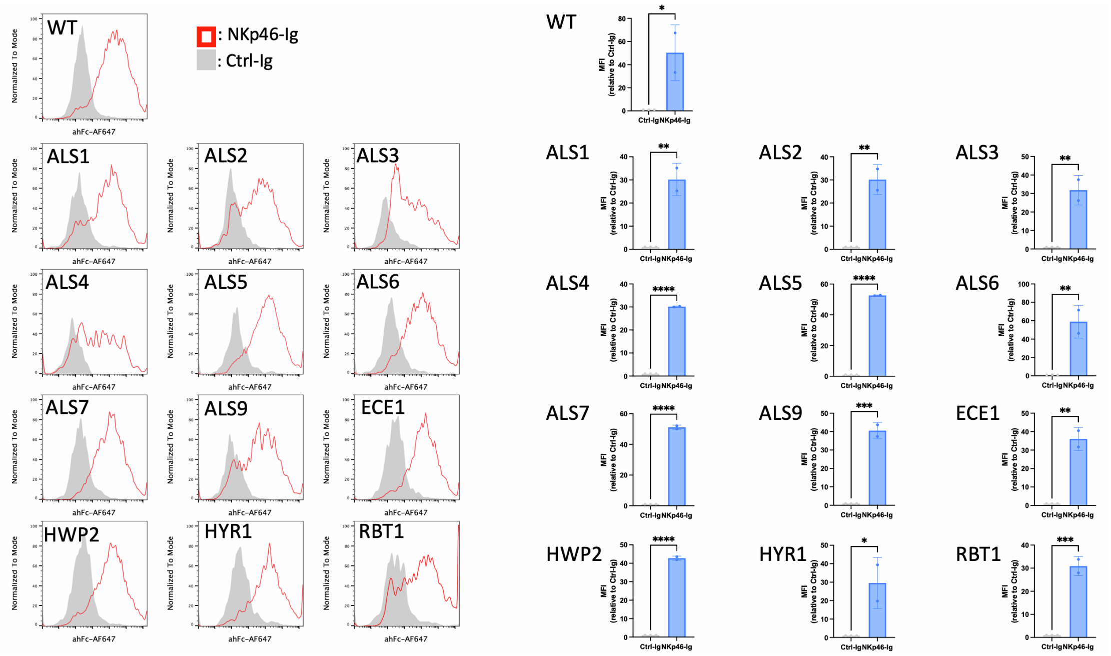

Supplementary Figure 4. Major adhesion molecules of *C. albicans* are not NKp46 ligands.

Related to Figure 2.

C. albicans strains lacking key adhesion molecules were stained with NKp46-Ig, followed by an anti-human Fc AF647-conjugated secondary antibody. Experiments were independently performed three twice, and the MFI values from each experiment were pooled for statistical analysis. Data are represented as mean +/- SD. Statistical significance was determined using an unpaired two-tailed Student's *t*-test ($P < 0.05$ was considered statistically significant). Statistical significance is indicated as follows: $P < 0.05$ (*), $P < 0.01$ (**), $P < 0.001$ (***), $P < 0.0001$ (****).

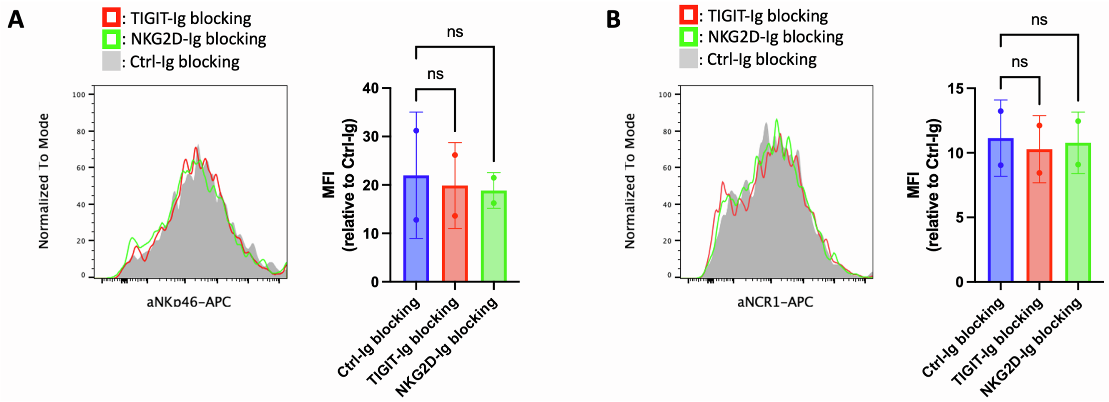

Supplementary Figure 5. NKG2D-Ig and TIGIT-Ig do not block NCR1-Ig or NKp46-Ig binding to C. albicans. Related to Figure 2.

(A) The hyphae of *C. albicans were* stained with NKG2D-Ig and TIGIT-Ig first, followed by NKp46-Ig and anti-NKp46 APC antibody. (B) The hyphae of *C. albicans were* stained with blocking fusion proteins first, followed by NCR1-Ig and anti-NCR1 APC antibody Experiments were independently performed twice, and the MFI values from each experiment were pooled for statistical analysis. Data are represented as mean +/- SD. Statistical analysis was performed using ordinary one-way ANOVA followed by Tukey’s multiple comparisons test.
